# Mechanistic Insight into the Reactivation of BCAII Enzyme from Denatured and Molten Globule States by Eukaryotic Ribosomes and Domain V rRNAs

**DOI:** 10.1371/journal.pone.0153928

**Published:** 2016-04-21

**Authors:** Biprashekhar Chakraborty, Sayan Bhakta, Jayati Sengupta

**Affiliations:** Structural Biology & Bio-Informatics Division, Indian Institute of Chemical Biology (Council of Scientific & Industrial Research), 4, Raja S.C. Mullick Road, Kolkata, 700 032, India; University of Lethbridge, CANADA

## Abstract

In all life forms, decoding of messenger-RNA into polypeptide chain is accomplished by the ribosome. Several protein chaperones are known to bind at the exit of ribosomal tunnel to ensure proper folding of the nascent chain by inhibiting their premature folding in the densely crowded environment of the cell. However, accumulating evidence suggests that ribosome may play a chaperone role in protein folding events *in vitro*. Ribosome-mediated folding of denatured proteins by prokaryotic ribosomes has been studied extensively. The RNA-assisted chaperone activity of the prokaryotic ribosome has been attributed to the domain V, a span of 23S rRNA at the intersubunit side of the large subunit encompassing the Peptidyl Transferase Centre. Evidently, this functional property of ribosome is unrelated to the nascent chain protein folding at the exit of the ribosomal tunnel. Here, we seek to scrutinize whether this unique function is conserved in a primitive kinetoplastid group of eukaryotic species *Leishmania donovani* where the ribosome structure possesses distinct additional features and appears markedly different compared to other higher eukaryotic ribosomes. Bovine Carbonic Anhydrase II (BCAII) enzyme was considered as the model protein. Our results manifest that domain V of the large subunit rRNA of Leishmania ribosomes preserves chaperone activity suggesting that ribosome-mediated protein folding is, indeed, a conserved phenomenon. Further, we aimed to investigate the mechanism underpinning the ribosome-assisted protein reactivation process. Interestingly, the surface plasmon resonance binding analyses exhibit that rRNA guides productive folding by directly interacting with molten globule-like states of the protein. In contrast, native protein shows no notable affinity to the rRNA. Thus, our study not only confirms conserved, RNA-mediated chaperoning role of ribosome but also provides crucial insight into the mechanism of the process.

## Introduction

Ribosome, a dynamic ribonucleoprotein complex (~2.4MDa in prokaryotes and ~4MDa in eukaryotes), is widely known to be the key player of protein synthesizing machinery of every living cell upon which the genetic code within the mRNA is translated into the primary amino acid sequence [1]. Most of the newly synthesized polypeptide chain must adopt native conformation, characteristic of each protein, to gain functional activity [2]. Several chaperones are known to bind ribosome to ensure proper folding of the nascent chain by inhibiting their premature folding in the densely crowded environment of the cell.

It has been evidenced that many proteins fold spontaneously *in vitro*, confirming Anfinsen’s pioneering proposition that the primary amino acid sequence of a protein determines its folding and given proper conditions, proteins can fold spontaneously either co-translationally or post-translationally [3]. Nevertheless, a line of defence against the misfolding and aggregation of the emerging polypeptide in the densely crowded cellular environment is present in both prokaryotes and eukaryotes in the form of molecular chaperones.

Considering ribosome biogenesis as one of the most energy consuming cellular processes [4], it is conceivable that this molecular machine have some auxiliary functional properties which are also utilized by the cell. In fact, over the past several years it has been proposed by different research groups that ribosome is the hub of protein biogenesis, controlling not only the protein biosynthesis but also the process of folding nascent polypeptides (reviewed in:[5–7]). A number of previous studies have also suggested co-translational model of folding for eukaryotes [8–10]. Luciferase, an enzyme as big as 62KDa, was found to show full activity upon translation in a cell free extract lacking chaperones [9]. This finding not only establishes the co-translation mode of folding but also hints towards an intrinsic chaperoning ability of the translating apparatus itself, as proposed by Alexander Spirin and co-authors [9]. Thus, although the preeminent function of the ribosome is translation of messenger RNA into polypeptides, growing evidence indicates that ribosome may play a pivotal role in the surveillance of proper folding of the nascent polypeptide chains as well.

Interestingly, several groups have reported ribosome’s ability to fold protein in a trans-acting manner [11–15]. Domain V of the large ribosomal RNA (rRNA) has been identified as the key player primarily responsible for this function [13–15]. Domain V forms a large canyon at the intersubunit side of the large subunit extending from L1 stalk to L7/L12 stalk, and it contains the active site for the formation of peptide bonds, the peptidyl-transferase centre (PTC) [16]. Seemingly, this chaperone property of ribosome is unrelated to the nascent polypeptide chain folding at the exit of ribosomal tunnel. Most of these studies have been done *in vitro* on prokaryotic systems proclaiming refolding ability of ribosome and its domain V RNA. However, limited studies with ribosomes and domain V RNA from some eukaryotic sources (yeast, wheat germ, rat liver, drosophila) also indicated protein folding ability of ribosomes [13, 15, 17, 18].

Here we have tried to explore the mechanism underlying RNA-based protein folding activity of the ribosome which still remains elusive. We have focussed on eukaryotic systems and considered a primitive eukaryotic species *Leishmania donovani*, along with the commonly studied eukaryotic species *Saccharomyces cerevisiae* (yeast) as model organisms. *L*. *donovani* is a member of the kinetoplastid family and like most of its family members it is responsible for causing pathogenic disease in human apart from the fact that this group of organisms have some unique characteristics. Firstly, this group of organisms hardly have got any regulation at the transcriptional level making translational regulation more vital [19]. Recent cryo-electron microscopy structure of *T*. *brucei* (a member of kinetoplastid group) ribosome [20] revealed that it is not only much more complex than that of its prokaryotic counterparts [16], the size and arrangement of the ribosomal RNA (rRNA) expansion segments are also markedly different as compared to other eukaryotes such as yeast ribosome [21]. Moreover, the large subunit rRNA of these species is also fragmented into a number of domains where each of them is coded by separate gene.

Together with published data, our results demonstrate a conserved nature of protein folding activity among prokaryotic and eukaryotic species. Also, consistent with the previous results, domain V of the large ribosomal RNA is identified as the potential candidate for this function in case of Leishmania ribosomes.

Our objective was not only to understand whether domain V RNA mediated protein folding is a general phenomenon in spite of the differences in overall ribosome structures, but also to gain insight into the mechanism of the process. To this end, we have characterized stable molten globule-like intermediates of Bovine Carbonic Anhydrase (BCAII) and investigated binding affinities of native, molten globule-like and denatured forms of the BCAII enzyme towards domain V of the eukaryotic large subunit rRNA using Surface Plasmon Resonance (SPR) technique. A recent study has demonstrated that *E*. *coli* ribosome can recover active protein from folding intermediates of BCAII [22]. In line with this study, our results also manifest that eukaryotic ribosomes (as well as domain V rRNAs) are able to rescue BCAII activity from the intermediate molten globule-like state. Our SPR experiments reveal that the domain V rRNA component has remarkable affinity specifically towards the molten globule-like states of the protein. This finding sheds light on the possible mechanism by which the ribosome acts as a protein folding modulator.

## Results

### Understanding the GuHCl-mediated denaturation pathway of BCAII in presence or absence of EDTA

**Fig 1:**
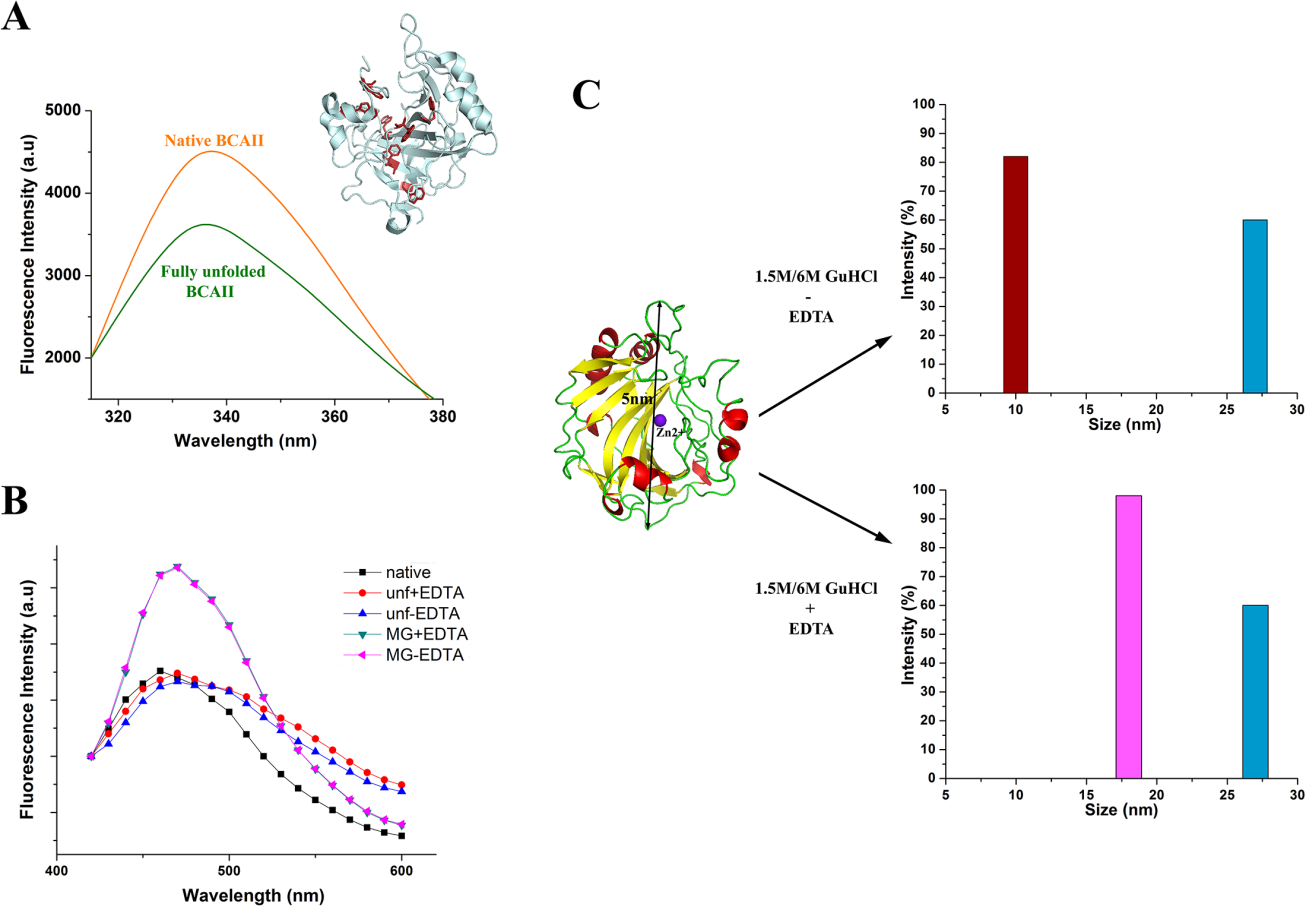
Denaturant-induced unfolding of BCAII. (**A)** Tryptophan fluorescence of native (orange) and denatured (green) Bovina Carbonic Anhydrase II (BCAII) shows reduction in fluorescence intensity upon denaturation. In the inset a cartoon representation of the crystal structure of BCAII enzyme (PDB code 1V9E) is shown with the tryptophan residues highlighted in red stick. **(B)** Emission spectra (320–600) of the extrinsic fluorescence of native, fully unfolded (unf+EDTA; unf-EDTA) and molten globule (MG+EDTA;MG-EDTA) BCAII recorded using 8-anilinonaphthalene-1-sulphonic acid (ANS) dye showing considerably higher binding of ANS with molten globule BCAII compared to native or unfolded BCAII. (**C)** Crystal structure of BCAII enzyme (PDB code 1V9E) having a diameter of ~5nm with its secondary structures highlighted in different colors. Under different conditions, change in the hydrodynamic diameter of native BCA upon denaturation to molten globule-like (~80% population 10nm in 1.5 M GuHCl without EDTA (brown), 100% population 18 nm in 1.5 M GuHCl with EDTA (pink)) and fully unfolded state (~60% population 27 nm in 6 M GuHCl with or without EDTA (blue)) is shown as obtained from dynamic light scattering (DLS) experiments (the experiment was repeated twice for each case, acquiring data twice each time).

First, we aimed to characterize the denatured and stable intermediate states of the Bovine Carbonic Anhydrase II (BCAII) enzyme (used as the model protein in this study) unfolding pathway and the role of EDTA in the process. It has been documented that mild denaturing condition (1.5 M GuHCl) produces molten globule-like intermediate state and higher concentration of GuHCl (~5–6 M) is required to achieve fully unfolded state for both BCAII and HCAII with BCAII requiring EDTA (for removing the Zn^+2^ ion) along with denaturant, unlike HCAII to produce these states [23–26]. Apparently, the difference in Zn^+2^ binding affinity is responsible for this differential unfolding behaviour of these two proteins despite their high sequence and structural similarities [23]. Unfolding of native BCAII by 6M GuHCl was confirmed by tryptophan fluorescence (Fig 1A).

Several studies have reported molten globule-like intermediate states of BCAII of different sizes [25, 27]. We tried to track down the different possible folding intermediates of BCAII using ANS fluorescence and dynamic light scattering (DLS) analysis of BCAII, denatured chemically by 6M GuHCl as well as 1.5M GuHCl (both in presence and absence of EDTA). The ANS fluorescence intensity for BCAII denatured with 1.5M GuHCl both in presence or absence of EDTA was found to be considerably higher than both native BCAII and the enzyme denatured with 6M GuHCl either in presence or in absence of EDTA (Fig 1B) confirming that denaturation of BCAII by 1.5M GuHCl results in the formation of a molten globule-like intermediates unlike denaturation by 6M GuHCl which results in complete unfolding. Further, DLS analysis showed that while BCAII denatured with 6M GuHCl irrespective of presence or absence of EDTA was found to be populated (~60%) by a particle with hydrodynamic diameter of about 27nm (Fig 1C)while BCAII denatured with low GuHCl concentration (1.5M) was found to be populated (~98%) by particles having a hydrodynamic diameter of about 18nm in presence of EDTA and about 10nm (~80%) in its absence (Fig 1C). The two intermediates (10 nm and 18nm) we observed correspond well with the ‘dry’ (when water has not penetrated into the protein core) and ‘wet’ (hydrated core) molten globules, previously characterized for GuHCl mediated protein denaturation [28, 29].

Our analysis, in line with previous observations, clearly demonstrates that BCAII enzyme can be denatured into different forms by modulating the denaturing mixture, specifically implying the crucial role played by EDTA in this regard.

### BCAII refolding by leishmania ribosome is slower as compared to its yeast counterpart

**Fig 2:**
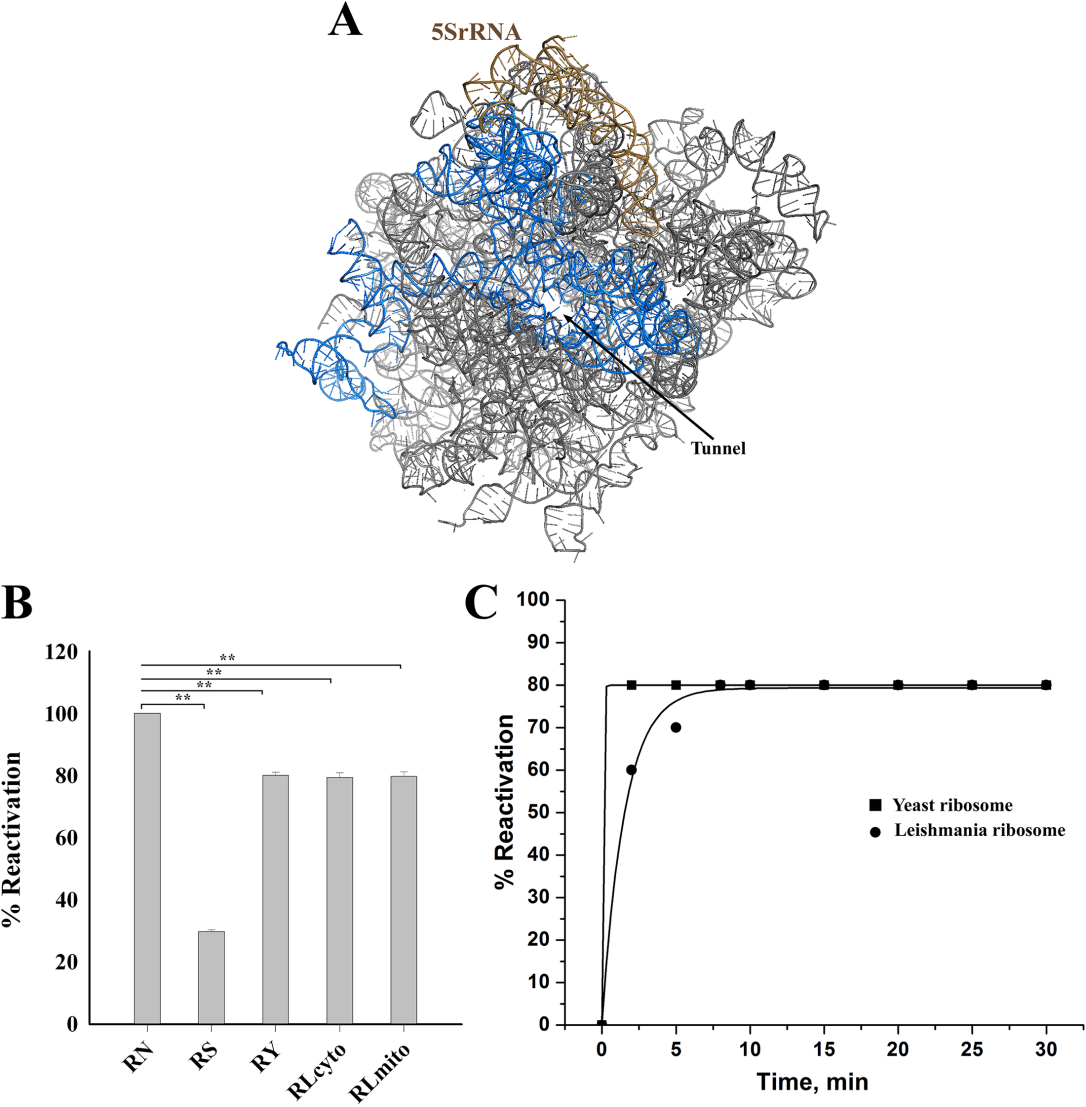
Refolding of denatured BCAII by eukaryotic ribosomes. (**A)** Intersubunit interface view of the large Subunit rRNA (grey) of *Saccharomyces cerevisiae* (PDB code 4V88) with the domain V (highlighted in blue) around the entrance of the peptide tunnel (marked). (**B)** Reactivation of fully denatured BCAII in presence of eukaryotic ribosomes from *Saccharomyces cerevisiae* (RY) and *Leishmania donovani* (both cytosolic (RL-cyto) and mitochondrial ribosome (RL-mito)) shows ~80% recovery of protein’s activity. RN shows the activity of native protein (considered as 100%), while RS represents self refolding of the denatured protein in presence of EDTA (~30%). Data are presented as means ± SEM. ** (p < 0.001, one-way ANOVA, N = 5) shows statistically significant differences from control (RN). (**C)** The rate of ribosome-assisted reactivation is faster for 80S ribosome (cytosolic) from *Saccharomyces cerevisiae* (▪) as compared to *Leishmania donovani* (•).

In order to examine the conservation of domain V RNA (Fig 2A) mediated chaperoning ability of ribosome among eukaryotes, retrieval of enzymatic activity of BCAII enzyme denatured by 6M GuHCl in presence of EDTA [30–32] was assayed following 30 minutes incubation in presence of 80S ribosomes from yeast and leishmania.

80S ribosomes from both *Leishmania donovani* (L80S) and *Saccharomyces cerevisiae* (Y80S) showed almost equal efficiency in refolding fully denatured BCAII retrieving about 80–85% of the protein’s enzymatic activity (Fig 2B). Additionally mitochondrial ribosome from *Leishmania donovani* was also tested for its protein folding ability and it was found to retrieve about 80% of activity (Fig 2B). Previous studies showed that *E*. *coli* ribosome can also refold denatured BCAII with similar efficiency [30]. This observation indicates that at least for small proteins like BCAII efficiency of folding remains almost the same irrespective of the source of ribosome.

We performed time-dependent assay for both L80S and Y80S to check the kinetic behaviour of their refolding activity. Interestingly, we observed that while Y80S refolded denatured BCAII so as to retrieve maximal enzymatic activity within 2 minutes, it took about 8 minutes by L80S to do the same (Fig 2C). It may be noted here that *E*.*coli* ribosome also takes about 8 minutes to fold BCAII in vitro [30].

### Domain V belongs to the beta subunit of Leishmania LSU rRNA

Previous studies have already identified the peptidyl transferase center (PTC) located in the domain V (576 nucleotides) of *E*.*coli* large subunit RNA as the primary chaperoning component of ribosome [33–35]. We aimed to investigate whether the same domain is responsible for catalysing protein folding in case of leishmania. First, we tried to map the RNA fragment of 28S rRNA of *L*. *donovani* harbouring domain V.

**Fig 3:**
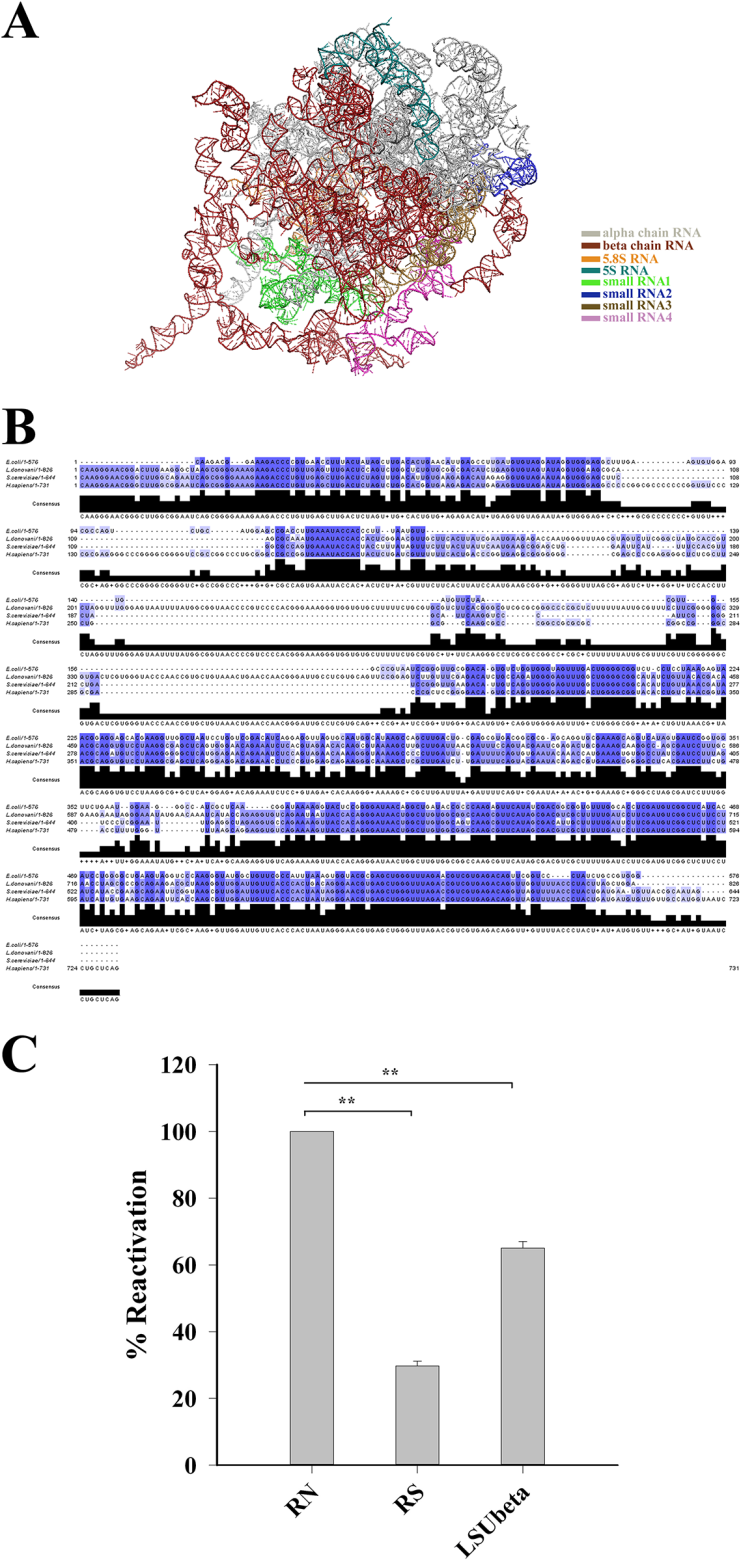
*L*. *donovani* LSUβ RNA-assisted folding of denatured BCAII. (**A)** Large subunit rRNA (LSU-RNA) of *Trypanosoma brucei* (PDB code 4V8M) viewed from intersubunit side with different RNA domains (marked in different colours) shows extra rRNA helices. (**B)** Alignment of the domain V rRNA sequence of *L*. *donovani* with the same from *E*. *coli*, *S*.*cerevisiae* and *human*. (**C**) LSUβ domain of *L*. *donovani* shows ~65% reactivation of fully denatured BCAII. RN and RS represent the same as Fig 1. Statistical significance is shown by ** (p < 0.001, one-way ANOVA, N = 5) compared to control (RN).

The uniqueness of all the trypanosomatid ribosomes, including Leishmnia, is that the large subunit (28S) rRNA chain is segmented into five pieces namely alpha (LSUα), beta (LSUβ), gamma (LSUγ), delta (LSUδ), epsilon (LSUε) etc., each coded by different genes (Fig 3A). Of all these, the alpha and the beta domains are quite large with about 1800 and 1500 nucleotides in length respectively. The rests are about 200 nucleotides or less in length.

However, no secondary structure for any of these RNA fragments of *L*. *donovani* is available. Considering the length of *E*.*coli* domain V RNA fragment we reasoned the *L*. *donovani* domain V RNA to be present either within LSUα or LSUβ fragment. Further, based on the structural similarity of the beta domain of *Trypanosoma brucei* (only trypanosomatid with rRNA secondary structure available (http://www.rna.ccbb.utexas.edu)) with that of *E*. *coli* and *S*. *cerevisiae* we hypothesized that the beta domain harbours the domain V RNA.

The LSUβ gene of *L*. *donovani* was cloned and subsequently sequenced to identify the domain V region within it. The *L*.*donovani* domain V RNA when aligned with the same from *E*.*coli*, *S*.*cerevisiae* and human, it showed ~60% sequence similarity with the *E*.*coli* domain V and ~77% sequence similarity with yeast and human counterparts (Fig 3B and S3 Table). The LSUβ RNA domain obtained upon run-off transcription was found to be modulating folding of denatured BCAII enzyme, retrieving about 65% of its activity upon 30 min of incubation (Fig 3C). Thus, it was confirmed (both structurally and functionally) that the LSUβ RNA domain of *L*. *donovani* harbours the domain V RNA.

### Domain V RNA of *Leishmania donovani* has got similar chaperoning ability as its bacterial and eukaryotic counterparts

Domain V of the large subunit rRNA of the ribosome is known to be the actual modulator for ribosome-mediated protein folding. Here we have tried to compare the ability of leishmania domain V RNA (LdV) towards the retrieval of enzymatic activity of the denatured protein with that of its yeast counterpart (YdV).

**Fig 4:**
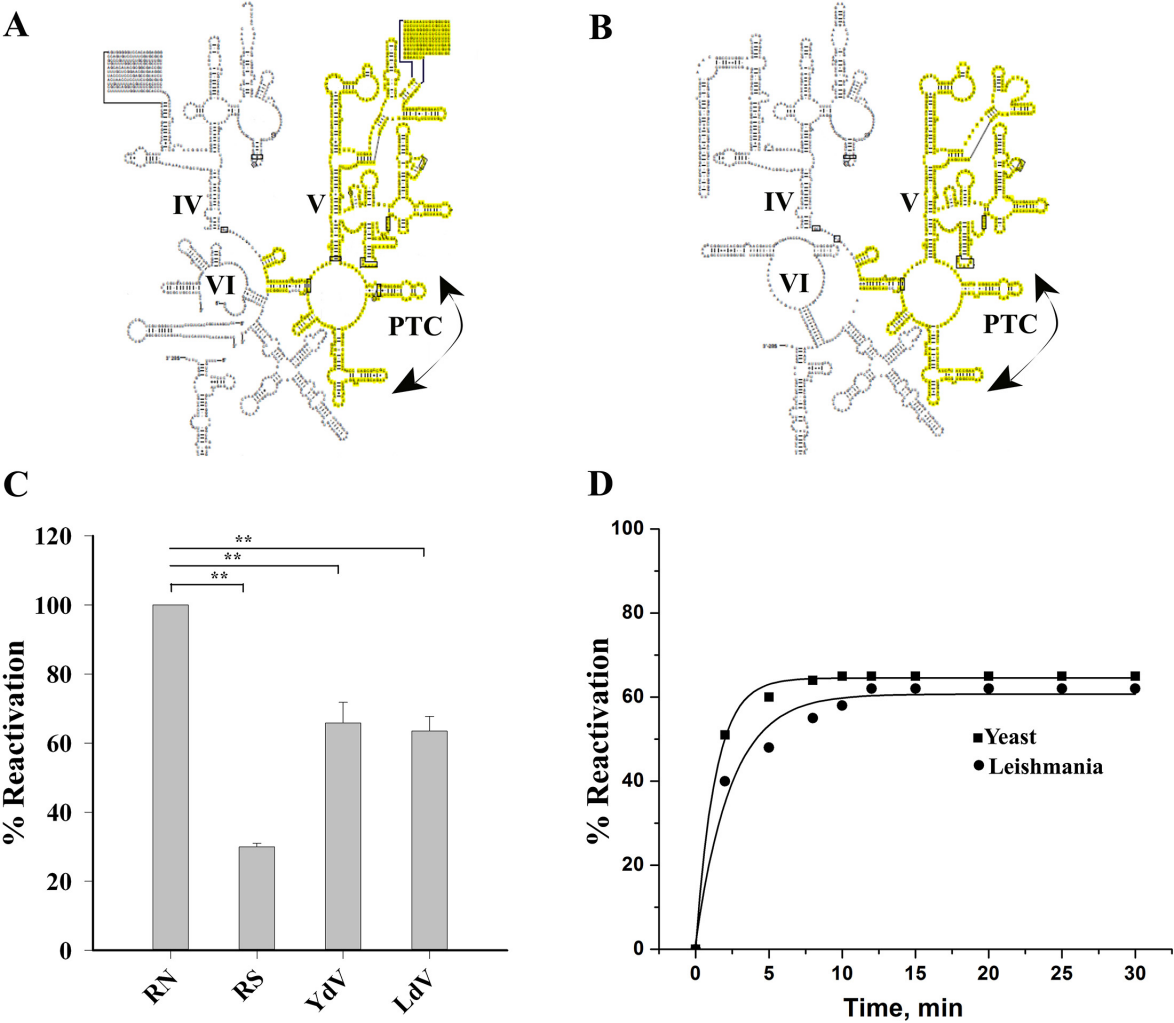
Eukaryotic ribosomal RNA domain V-mediated folding of denatured BCAII. Secondary structure diagram of 3’end of the LSU-RNA of *T*. *brucei* with the domain V (highlighted in yellow) **(A)** and the same of *S*. *cerevisiae* **(B)**. The peptidyl transferase centre (PTC) in domain V is marked with arrows in (A and B). (**C)** BCAII reactivation by the domain V RNA of *L*. *donovani* (LdV) and *S*. *cerevisiae* (YdV) shows ~65% activity recovered. RN and RS represent the same as in Fig 1. Statistical significance is shown by ** (p < 0.001, one-way ANOVA, N = 5) compared to control (RN). (**D)** Time course of reactivation of BCAII by domain V RNA from *S*. *cerevisiae* (▪) and *L*. *donovani* (•) shows faster reactivation for *S*. *cerevisiae*.

Domain V region of LSUβ fragment of *L*. *donovani* and domain V of Y80S large subunit rRNA were cloned for this purpose. Domain V RNA component from both yeast (YdV) and leishmnia (LdV) (Fig 4A and 4B), like their prokaryotic counterpart [35], were found to retrieve about 65% of BCAII activity (Fig 4C). This finding confirms that domain V rRNA is, indeed, responsible for the intrinsic protein chaperone activity shown by the ribosome. Thus, domain V rRNA can be considered as the universal chaperoning component of the ribosome.

The time course of both YdV and LdV mediated BCAII folding was performed and it was found (Fig 4D) that YdV folds BCAII faster than LdV. This data is in good agreement with the trends observed for the ribosome-mediated folding rates of the two species. While YdV takes about 8 min for the maximal BCAII folding, it takes LdV about 12 min to do the same (similar to that of the domain V from *E*.*coli* [31]).

### Eukaryotic ribosomes and their domain V rRNAs can rescue a protein from molten globule-like state

Next, we wanted to investigate whether eukaryotic ribosome and its domain V rRNA can retrieve functional protein from partially-folded, molten globule-like states in the same way as their bacterial counterpart [22]. The molten globule-like state of BCAII obtained upon partial denaturation (1.5M GuHCl) in absence of EDTA was found to get self refolded with great efficiency (~65%) unlike when denatured with 1.5M GuHCl in presence of EDTA (~30%) as suggested in previous studies also [23]. So, the less compact molten globule-like intermediate of BCAII obtained in presence of EDTA was selected to test the effect of 80S ribosome as well as eukaryotic domain-V rRNA towards its folding.

**Fig 5:**
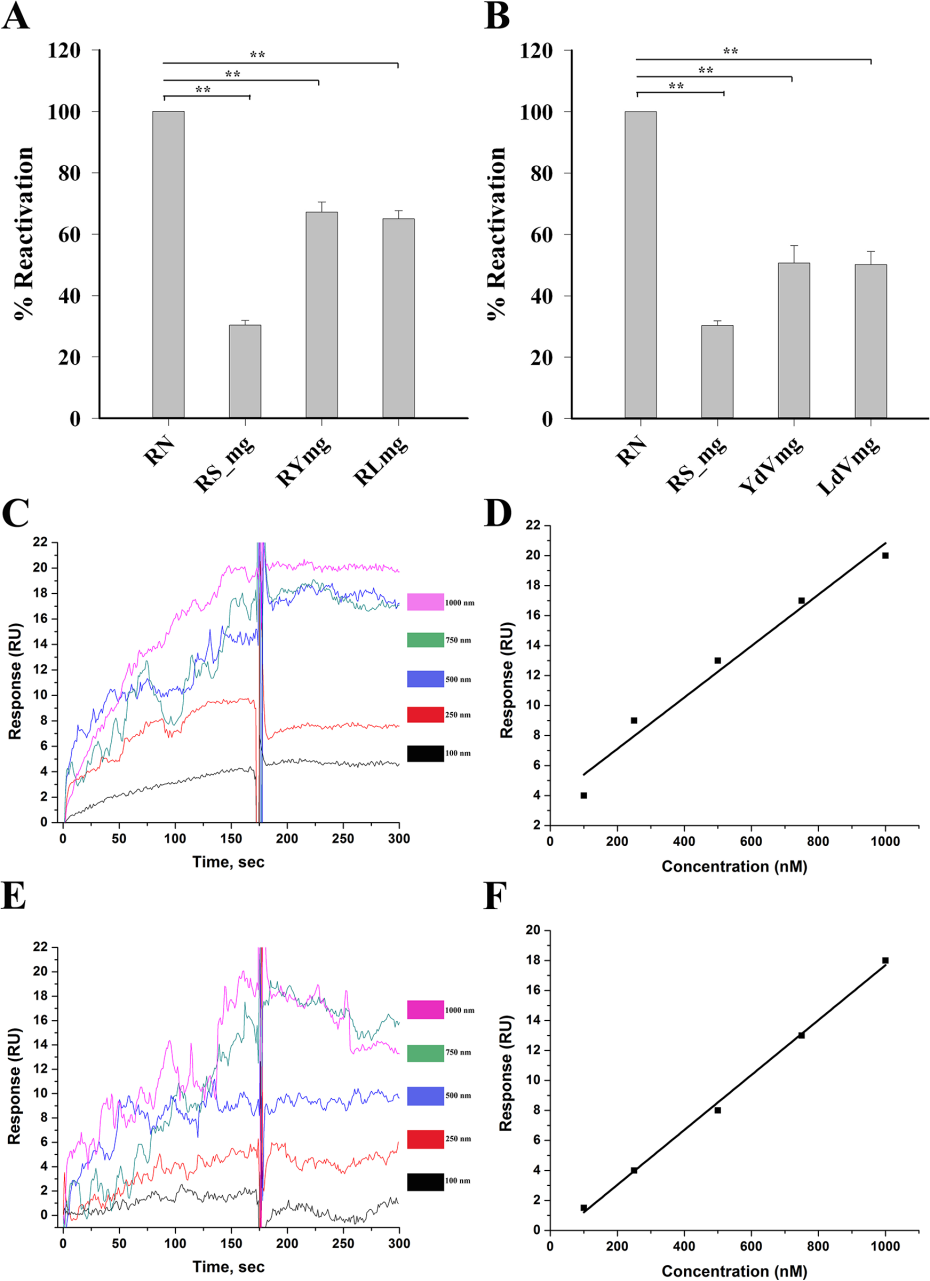
The effect of eukaryotic ribosomes and their domain V rRNA over MG-like states of BCAII. (**A)** The *S*. *cerevisiae* (RYmg) and *L*. *donovani* (RLmg) 80S ribosome-assisted reactivation of molten globule-like (MG-like) BCAII suggests ~60–65% recovery. (**B)** Domain V RNA of *S*. *cerevisiae* (YdVmg) and *L*. *donovani* (LdVmg) reactivates 50–55% BCAII from MG-like state. RN represents the same as in Fig 1 while RS_mg represents self refolding of the MG-like protein in presence of EDTA (~30%). Statistical significance is shown by ** (p < 0.001, one-way ANOVA, N = 5) compared to control. (**C-D**) Interaction of MG-like state of BCAII protein with the domain V rRNA from *L*.*donovani* and *S*.*cerevisiae* examined by SPR analysis is shown respectively. Sensograms recorded when varying concentrations of protein (in MG-like state) were passed over the RNA immobilized on the streptavidin chip showing the binding profile of MG-like BCAII with LdV **(C)** and YdV (**E**). The interaction pattern suggests significant affinity of the protein in MG-like state towards domain V RNA. Concentration dependent increase in protein (MG-BCAII) binding is shown for LdV (**D**), and YdV (**F**).

80S ribosomes from both yeast and leishmania (Y80S and L80S) were shown to retrieve about 60–65% of BCAII activity (Fig 5A) while their corresponding domain V rRNAs retrieved about 50–55% of the enzyme activity (Fig 5B).

This observation also confirmed that the particles having ~18nm hydrodynamic diameter are not aggregates, but represent partially-unfolded (more open) intermediate form. Thus, ribosome and the rRNA fragment responsible for its folding activity can rescue proteins trapped in intermediate states to a significant extent so as to prevent them from going towards aggregation.

### BCAII in molten globule-like state binds domain V rRNA with considerable affinity

The domain V rRNA being the primary chaperoning component of ribosome, it is expected to interact with its substrates during the course of folding. In order to understand the kinetics of interaction between the rRNA and BCAII enzyme, we monitored interactions between the immobilized domain V rRNA with native, molten globule-like (denaturation under 1.5M GuHCl in presence of EDTA) and denatured BCAII (with 6M GuHCl) individually using surface plasmon resonance (SPR) technique.

**Fig 6:**
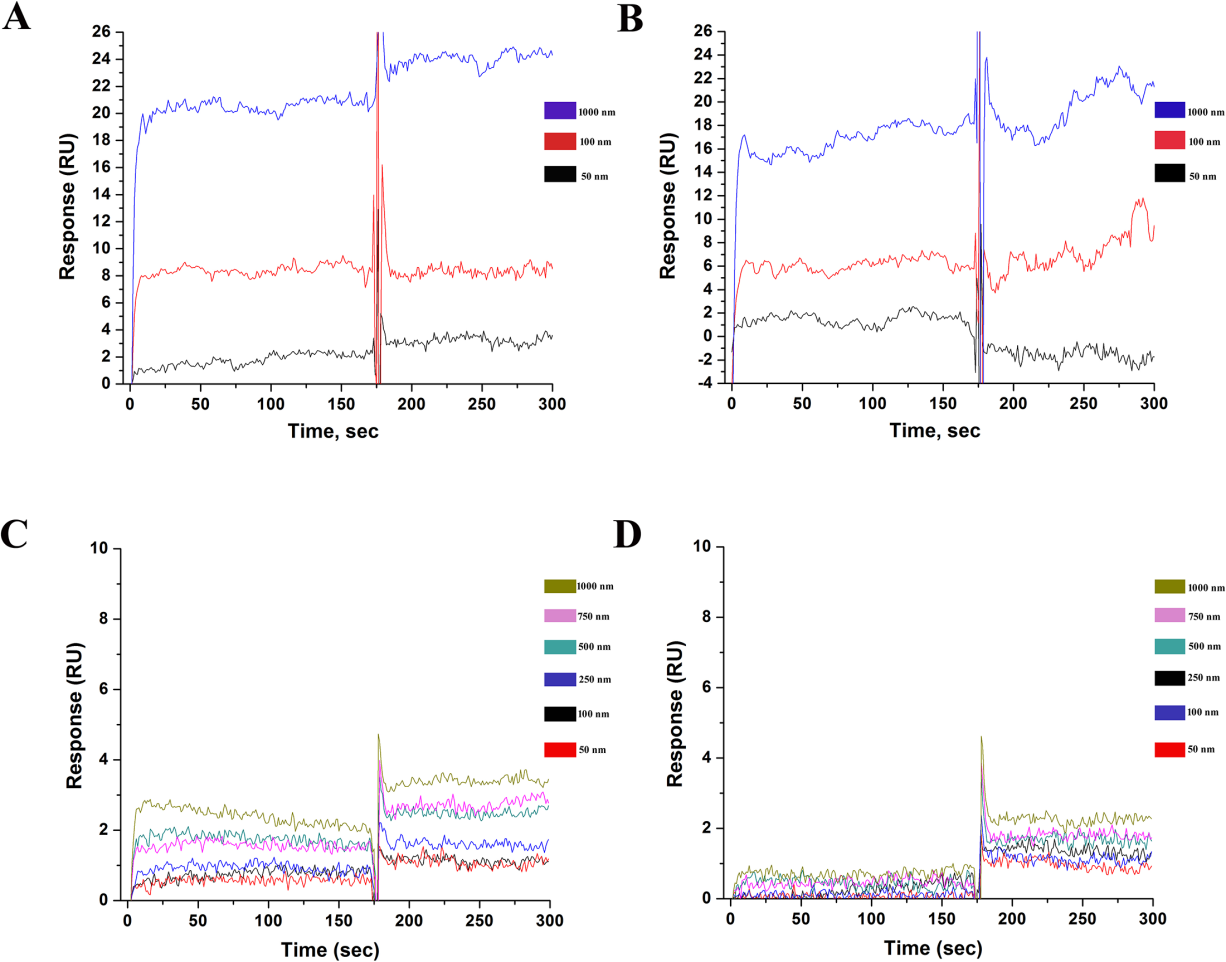
Binding analysis of BCAII in denatured and native states with the eukaryotic rRNA. Sensogram recorded when varying concentrations of protein (in fully denatured state (**A**) and native state (**B**)) were injected over the RNA immobilized on the streptavidin chip. Binding profile of fully denatured BCAII with LdV (**A**) and YdV (**B**) shows unusually steep association pattern indicating very fast mode of interaction. The experiment with denatured protein was done in low temperature. No notable concentration dependent increase in binding is observed for native BCAII with either LdV (**C**) or YdV (**D**) indicating no significant affinity of the native protein towards RNA.

Under room temperature (25°C) BCAII in partially denatured, molten globule-like state showed considerable binding affinity towards domain V rRNA from both the species (YdV, LdV) (Fig 5C–5F) but neither native BCAII (Fig 6C and 6D) nor its fully denatured form showed any respectable affinity for the same. It was presumed that interaction between domain V rRNA and the fully unfolded protein couldn’t be detected due to very fast and transient nature of the interaction (as a result of high entropy of the fully unfolded protein). Indeed, interaction between the domain V RNAs and fully denatured BCAII was observed (Fig 6A and 6B) when the experiment was done at 15°C (denatured protein in chilled sample buffer).

Interestingly, the association profiles obtained for molten globule-like (Fig 5C and 5E) and fully denatured BCAII (Fig 6A and 6B) with the rRNA were found to be distinctly different indicating variance in the mode of interaction of rRNA with the two forms of BCAII. The steep pattern of the association profile obtained for denatured BCAII-rRNA interactions indicate that the fully unfolded protein makes some very fast initial contacts with domain V RNA (even at 15°C, reaching saturation within about 15–20 seconds (Fig 6A and 6B)). In contrast, comparatively slow and steady association profile demonstrates specific interactions with domain V rRNA stabilize the molten globule-like state of BCAII (Fig 5C and 5E). Collectively, we propose that the RNA cage of the ribosome restricts the entropy of the fully unfolded protein so that it descend into the more structured molten globule-like state very quickly and then the specific RNA-protein interactions come into play in order to drive molten globules towards proper folding into its native state.

## Discussion

Substantial evidences have been accumulated suggesting that RNA can function as molecular chaperone [5, 7, 12–14, 17, 18, 30, 32–39]. The 50S subunit specific antibiotic chloramphenicol, which binds at the PTC and efficiently stops protein synthesis, also inhibits protein folding activity of the ribosome suggesting the domain V as the functional site for the chaperone activity as well [40]. Our data corroborate the previous results and showed that protein folding ability of ribosome as well as domain V rRNA is conserved across different eukaryotic species also.

In accordance with the crystallographic studies the conserved domain V encompassing PTC is mostly buried in the intersubunit face of the large subunit [16, 21, 41]. Thus, mechanistically it is difficult to conceive how domain V can assist protein folding in a trans-acting manner. Since ribosome can recover BCAII activity substantially from molten globule-like state also, it is evident that not only the denatured protein, but also a protein trapped in some intermediate state of folding can access domain V of the ribosome in a trans-acting manner. Recent studies have assigned some nucleotides at the PTC of the *E*. *coli* 70S ribosome which are involved in the protein folding activity [17, 32, 37]. When we mapped the nucleotides (mostly belong to the helices H89, H92 of the 23S rRNA) on the ribosome structure we found that these helices are located adjacent to the L7/L12 stalk base which remain partially exposed (S1 Fig) and can be accessed from the tRNA-entry side of the intersubunit space. It may be noted here that recent studies have identified the nucleotides responsible for the chaperone activity of domain V, and those nucleotides are mostly conserved across species (S1 and S2 Tables). Despite the differences in the overall large subunit structure, the 3D structures of domain V rRNA from different species also showed almost identical folding of this region (S1 Fig). Thus the mechanistic basis of the faster rate of refolding observed for denatured BCAII by the yeast ribosome (and its rRNA) compared to its bacterial or kinetoplastid counterpart is unclear.

We observed that folding capacity of domain V alone is not as efficient as the ribosome as a whole (also shown by earlier studies) [17]. One plausible explanation would be that domain V does not achieve correctly folded conformation in isolation. Ribosome most likely provides the ideal conformational state of domain V rRNA that is capable for assisting proper folding of a denatured protein to attain its fully functional form. The higher efficiency of the folding process by the ribosome as compared to the isolated domain V rRNA, may be due to the fact that intersubunit space of the ribosome provides partial steric confinement in addition to restricting degrees of freedom.

The mode of interactions between domain V rRNA and BCAII in native, denatured and molten globule states observed in SPR analysis provides an important insight into the mechanism of the RNA-mediated protein folding activity of ribosome. Molten globules are intermediate, native-like but less compact conformational states in the folding trajectory of proteins, and it is considered to be crucial for initializing the folding process [42]. These intermediates are formed during protein folding not only *in vitro* but also *in vivo*. The study of the folding of LDL receptor *in vivo* evidenced for the formation of non-native structure that appears to inhibit aggregation prior to the folding of the protein to its native form [43]. The molten globule intermediates are considered to be the most vulnerable and aggregation-prone state in the course of protein folding process and determine whether the protein goes towards misfolding and aggregation or folds properly [44].

From our observation it seemed that the denatured protein by virtue of its intrinsic entropy initially makes some fast interactions with the rRNA, but specific RNA-protein association takes place explicitly when the protein reaches more compact molten globule-like state. Thus, the key chaperone component of the ribosome stabilizes the crucial protein folding intermediate so as to promote proper folding. Very fast as well as transient interactions which were found to take place between the fully denatured protein and the RNA likely allows the disordered protein to transform into partially-folded form by providing kinetic stability and consequently lowering its entropy as suggested for GroEL-assisted protein folding (reviewed in [45]). Importantly, the domain V rRNA showed no significant affinity for the native enzyme suggesting that the successive substrate binding and release cycle, characteristic molecular mechanism of chaperone activity, exists for ribosome-assisted protein folding as well.

In a previous study, chaperone-like activity of 70S ribosome was attributed to its significant surface hydrophobicity [46]. Although the prevailing view is that molecular chaperone stabilizes substrate by hydrophobic shielding either *via* direct contact or encapsulation, recent evidence showed that charge as well as steric hindrance also act as important factors [5]. The RNA-protein (molten globule-like state) interaction patterns displayed in our study indicate that the binding most likely occurs by virtue of some non-covalent interactions between the protein and RNA in a specific manner, unlike hydrophobic interaction-mediated recognition. Consistent with this postulation, the RNA-protein interactions, mapped for domain V of 23S *E*. *coli* rRNA with BCAII in a recent study [39], revealed that not only nonpolar but also positively charged amino acids of the protein are involved. Interestingly, the interacting residues were identified in the unstructured loop regions on 3D structure of the proteins. It is known that stability of the loop regions is significantly low in molten globule states (reviewed in [47]). We speculate that direct interactions with the RNA restrict the degrees of freedom of the loop regions and there by prevent misfolding events.

Although the protein folding activity of ribosome has been studied mostly *in vitro*, it is tempting to surmise that such a universally conserved ribozyme action of protein folding must be utilized by the cell. Indeed, ribosome has been shown to act as a solubility enhancer to its surface-linked aggregation-prone proteins [38]. Furthermore, a recent study revealed that increase in large subunit ratio increases yields of functional recombinant proteins [48]. Chaperone activity of ribosome as a *bona-fide* cellular function may have significant consequence for protein folding *in vivo*. Our *in-vitro* observations on reactivation of a protein from its molten globule state by ribosome might be a hint that ribosome-mediated protein folding can be instrumental in rescuing proteins trapped in some intermediate stages of folding from going towards misfolding and aggregation during some adverse cellular conditions such as cellular stress.

## Materials and Methods

### Isolation of cytosolic ribosome from *S*. *cerevisiae*

A diploid wild type strain MATaα derived by combining two haploid strains 8534-10A (MATa, leu2, ura3, his4) and 6460-8D (MATα, met3) of *S*. *cerevisiae* was grown overnight in Yeast-Peptone-Dextrose (Y.P.D) complete medium at 30°C as described earlier [49]. This yeast strain required approximately 18 hours to reach log phase. Once log phase was reached (OD_595_ ~ 2.5) cells were kept at 4°C for at least 1 hour and then harvested at 6000 ×g for 10 minutes at 4°C. Yeast cell pellets were stored at -80°C till use. Ribosome from yeast was isolated following the protocol of purification of salt washed yeast 80S ribosomes as described by *Algire et*.*al* [50] with slight modifications as and when required. Briefly, cell pellets were re-suspended in ribosome buffer (100mM KOAc, 20mM HEPES-KOH, pH 7.6, 10mM Mg (OAc)_2_, 1mg/ml heparin, 2mM DTT, 0.5mM PMSF) and lysed by passage twice through a French pressure cell. The resulting lysate was first centrifuged at 17000 × g for 30 min at 4°C. The supernatant was carefully removed and centrifuged again at 350,000 × g for 1hr at 4°C. The supernatant was discarded and the pellet was washed with minimum volume (~1ml) of ribosome buffer and was finally resuspended in about 18 ml high salt buffer (Ribosome buffer plus 500 mM KCl) and kept in ice for 60 min with gentle stirring. Following high salt wash the solution was centrifuged at 16000 × g for 10 min at 4°C for 3 to 4 times till no pellet could be visible. The supernatant was then layered over a sucrose cushion and centrifuged at 350000 × g for 1 hr at 4°C. Following centrifugation, the supernatant was discarded and the final ribosome pellet was dissolved in storage buffer (10mM Tris-HCl (pH 7.5), 12.5mM Mg (OAc)_2_, 80mM KCl, 5mM 2-Mercaptoethanol, 0.5mM PMSF) and kept at -80°C.

### Isolation of cytosolic and mitochondrial ribosome from *L*. *donovani* promastigotes

*L*. *donovani* (UR6 strain) promastigotes were cultured in M-199 medium supplemented with 10% heat inactivated FBS. The pH of the medium was adjusted to 7.2. The parasites in culture were monitored regularly under light microscope. Culture was maintained by transferring the late log phase parasites into fresh medium at 1:10 dilution. Parasites in log phase were harvested by centrifugation at 2500 g for 15 min and washed twice in cold phosphate-buffered saline (PBS). Before harvesting the parasites, the culture was checked for any kind of contamination by examination under light microscope. The basic procedure followed for isolation of both cytosolic and mitochondrial ribosome from *Leishmania donovani* is same as that followed for yeast with some obvious modifications. Isolation of cytosolic ribosome from Leishmania is as follows: the Leishmania parasites were lysed by resuspending the cells in cold lysis buffer (50 mM HEPES-KOH [pH 7.6], 300 mM KCl, 10 mM Mg(OAc)_2_, 0.2 mM EDTA, 1mM 2-Mercaptoethanol, 0.5 mM PMSF, 20% Glycerol, 0.1% Triton X-100) for 5 min [51]. After 5 min the cells were centrifuged at 1000 g for 15 min to remove cell debris. Supernatant was again centrifuged at 12000 g for 20 min at 4°C. The pellet (P12) obtained is the mitochondrial pellet. The supernatant was again centrifuged at 30,000 g for 30 min and pellet was discarded. The supernatant was diluted with ribosome buffer (composition same as in case of yeast) and same protocol as yeast ribosome purification was followed.

The mitochondrial pellet (P12) obtained was re-suspended in ribosome buffer (composition same as in case of yeast). The suspension was sonicated for about 2 min at maximal intensity, centrifuged at 30,000 g for 30 min and further processed as in case of the cytosolic ribosomes from yeast and leishmania.

### Cloning and sequencing of the β-domain of large subunit rRNA (LSU-β) of *L*. *donovani*

The gene corresponding to the LSUβ domain of *Leishmania donovani* was isolated from the total RNA pool using Superscript One-step RT-PCR Kit (Invitrogen) and was subsequently cloned into PET28a vector. The primer sequences used are as follows:

Forward primer: 5’- ATA AGG ATC CCC CAA CTG CAG ACC -3’

Reverse primer: 5’ - ATA AGA ATT CTG GTG ATG AGC GTA CCA AG -3’.

It must be added over here that the full sequence of *Leishmania donovani* LSU-β gene was not available and so primers were designed partially based on the LSU-β gene sequence of closely related *Leishmania major*. The cloned LSU β gene was subsequently sequenced (Xcelris Labs Ltd., Ahmedabad, India).

### Cloning of the gene segment corresponding to domain V rRNA of *L*. *donovani* and *S*. *cerevisiae*

Having obtained the full gene sequence of the β subunit of the large subunit RNA (LSUβ) of *L*. *donovani*, primers were designed to clone specifically the gene segment corresponding to the domain V RNA (LdV). The primer sequences are as follows:

Forward primer: 5’-ATAAGGATCCCAAGGGAACGGAC-3’

Reverse primer: 5’-ATAAGAATTCTCCAGCTAAGTAGGGTAAAAC-3’

The gene segment corresponding to the domain V RNA fragment of *S*. *cerevisiae* (YdV) was also cloned. The primer sequences are as follows:

Forward primer: 5’-ATAAGGATCCCAAGGGAACGGG-3’

Reverse primer: 5’-ATAAAAGCTTCTATTGCGGTAACATTCATC-3’

The gene segments corresponding to the PTC-RNAs from both the species were subsequently cloned into PET28a vector.

### In vitro synthesis of LSU-β rRNA from *Leishmania donovani* and domain V rRNA from both *L*. *donovani* and *S*. *cerevisiae*

The LSUβ rRNA gene as well as the LdV and YdV gene segment cloned within PET28a vector under T7 promoter was in vitro transcribed to obtain the corresponding RNA as described earlier [31]. In each case the plasmid was first linearized and in vitro transcription was performed using 1 μg of linearized plasmid as template and T7 RNA Polymerase in a reaction volume of 100ul containing 5x transcription buffer, rNTP mix, DTT and made upto final volume using nuclease free water. The reaction mixture was incubated at 37°C for 2.5 hours. After that DNaseI was added to the reaction mixture and further incubated for another 20 mins (5 μl/100μl reaction mix). The synthesized RNA obtained was precipitated with salt and ethanol.

### Denaturation and subsequent refolding of Bovine Carbonic Anhydrase II (BCAII) in presence and absence of ribosome and ribosomal rRNA

Bovine Carbonic Anhydrase II (BCAII; procured from Sigma-Aldrich), the model protein used in this study to analyze ribosome and ribosomal RNA mediated folding was denatured and subsequently refolded as described earlier [31]. Briefly, 25 μM BCAII was chemically denatured with various concentrations of guanidine hydrochloride (GuHCl) in presence of 3.5 mM EDTA for 3 hr. While the BCAII denatured with 1.5 M GuHCl attained molten globule state [22, 23], the one denatured with 6 M GuHCl attained fully unfolded state[32]. The denatured protein was then diluted 100 times (final concentration 250 nM) in refolding buffer (50 mM Tris-HCl pH 7.5, 100 mM NaCl and 10 mM MgCl_2_ with or without ribosome or rRNA and incubated for 30 min at 25°C to allow folding. The residual amount of guanidine hydrochloride had no effect on the activities of the enzymes.

### Measurement of BCAII activity

The activity of refolded proteins was determined following the kinetics for 2 min at 420 nm on addition of Para-Nitro Phenyl Acetate (PNPA), the substrate of BCAII enzyme, to the refolding mixtures and expressed as percent of activity of the same amount of native protein. The concentration of ribosome and ribosomal RNA was equimolar to that of the protein.

Statistical Analyses were done using SigmaPlot (Systat Software, Inc., San Jose, CA, USA). Error bars represent the standard error of the mean (SEM). The levels of significance were calculated by performing one-way analysis of variance (one-way ANOVA). A *p* value less than 0.05 (*p* < 0.05) was taken as statistically significant (N = 5).

### Dynamic Light Scattering (DLS) measurements

Dynamic light scattering experiments were performed in MALVERN Nano-ZS system to confirm the fully unfolded and molten globule-like states of BCAII. Each experiment was repeated at least three times and the mean of all the values was considered as the final value in each case. BCAII was chemically denatured to both fully unfolded and molten globule form as described earlier both in presence and absence of EDTA.

### Binding analysis using Surface Plasmon Resonance (SPR)

Interaction between domain V RNA and Bovine Carbonic Anhydrase (BCA) was monitored using Surface Plasmon Resonance (SPR) in a BIACORE3000 instrument using Streptavidin (SA) chips (BIACORE). Briefly, domain V RNA (from both *Leishmania donovani* & *Saccharomyces cerevisiae*) was first biotinylated using 3’RNA Biotinylation Kit (Thermo Pierce) and subsequently injected over the SA chip for immobilization of the biotinylated RNA on the chip. RNA immobilization over the chip was indicated by the increase in the response unit (RU). RNA was immobilized until a RU in the range of 125–195 was reached. For interaction analysis with BCAII (native, fully denatured and molten globule), different concentrations of the protein (50nM-1000nM) were injected one after another over the immobilized RNA at a flow rate of 30μl/min and the association and dissociation of the protein was recorded. BCAII was denatured to fully unfolded and molten globule form as described earlier. Every time a buffer solution with 0 nM BCAII was included as blank. Refolding buffer (50 mM Tris-HCl pH 7.5, 100 mM NaCl and 10 mM MgCl_2_) was used as running and sample buffer in all the experiments.

After association and dissociation cycle of each BCAII concentration the surface was regenerated with two 30 seconds injections of 10mM HEPES-1M NaCl, pH 7.5 at a flow rate of 30μl/min. While in case of native and molten globule BCAII, SPR was performed at 25°C, for fully denatured BCAII the same was performed at 15°C. It may be noted here that each kinetics experiment was carried out using two flow channels (FC), one without the RNA (FC1) and another with the RNA (FC2) and the specific RNA-Protein interaction was evaluated by subtracting the reference surface signal (FC1) from the sensor surface signal (FC2) using BIAEvaluation 4.1 software. In case of the fully denatured and molten globule BCAII, the sample buffer contained some amount of the denaturants (GuHCl, EDTA) along with the protein. Signal from nonspecific interactions of denaturants with the RNA was also considered to get rid of the ‘bulk effect’. The experiment was repeated thrice for each case.

### Intrinsic and extrinsic fluorescence

Intrinsic or tryptophan (Trp) fluorescence was done using a Hitachi F7000 spectrofluorometer while extrinsic or ANS (8-anilinonaphthalene-1-sulphonic acid) fluorescence was performed in Hitachi F2700 with an excitation and emission slit of 5 mm in both the case. For Trp fluorescence excitation wavelength was set at 295 nm and excitation from 300 nm to 500 nm was recorded while for ANS fluorescence excitation wavelength was set at 380 nm and excitation for protein-ANS complex was measured from 420nm to 600nm. All fluorescence measurements were performed at room temperature and the final concentration of BCAII was kept 1μM every time.

## Supporting Information

S1 Graphical Abstract During ribosome-mediated protein folding the domain V rRNA specifically interacts with the molten globule-like state to ensure proper folding. (TIF)

S1 Table Previous mutational studies done on *E*. *coli* domain V rRNA to identify the bases responsible for the RNA-mediated folding activity of ribosome [32, 17; Das D *et al*. *J Biol Chem* 287: 37508–37521 (2012), Pang Y *et al*. *J Biol Chem* 288: 19081–19089 (2013)] and corresponding bases in the PTC regions of *L*. *donovani* and S. *cerevisiae* (the nucleotides different in other species compared to *E*.*coli* are marked in bold). (PDF)

S2 Table Previous additional mutational studies done on yeast domain V rRNA [17; Pang Y *et al*. *J Biol Chem* 288: 19081–19089 (2013)] which showed involvement in the protein folding activity (the nucleotide different in *E*.*coli* compared to other species is marked in bold). (PDF)

S3 Table Percent Identity Matrix (Created by Clustal 2.1) showing *L*. *donovani* domain V rRNA similar sequence identity with *S*. *cerevisiae and H*. *Sapiens domain V*, where as less identity with *E*. *coli* domain V rRNA. (PDF)

S1 Fig Structural information on Domain V rRNA. **(A)** *E*. *coli* 70S crystal structures (PDB codes: 2I2U, 2I2V) showing domain V of 23S rRNA in brown colour, and nucleotides responsible for denatured protein binding as identified in [Das A *et al*. *J Biol Chem* 286: 43771-43781(2011)] are coloured in navy blue. Entrance of the polypeptide tunnel is marked. **(B)** Available Domain V structures from different organisms (PDB codes: *E*. *coli*, 2I2V; *S*. *cerevisiae*, 4V88; *T*. *brucei*, 4V8M) superimposed showing very less deviation (RMSD < 5Å). Land marks: CP, central protuberance; SB, P-proteins stalk base.
(PDF)
