# Supplementary figures and images for: Mechanistic Insight into the Reactivation of BCAII Enzyme from Denatured and Molten Globule States by Eukaryotic Ribosomes and Domain V rRNAs
Source: PLoS One. 2016 Apr 21;11(4):e0153928. doi: 10.1371/journal.pone.0153928 (PMC4839638; doi:10.1371/journal.pone.0153928)

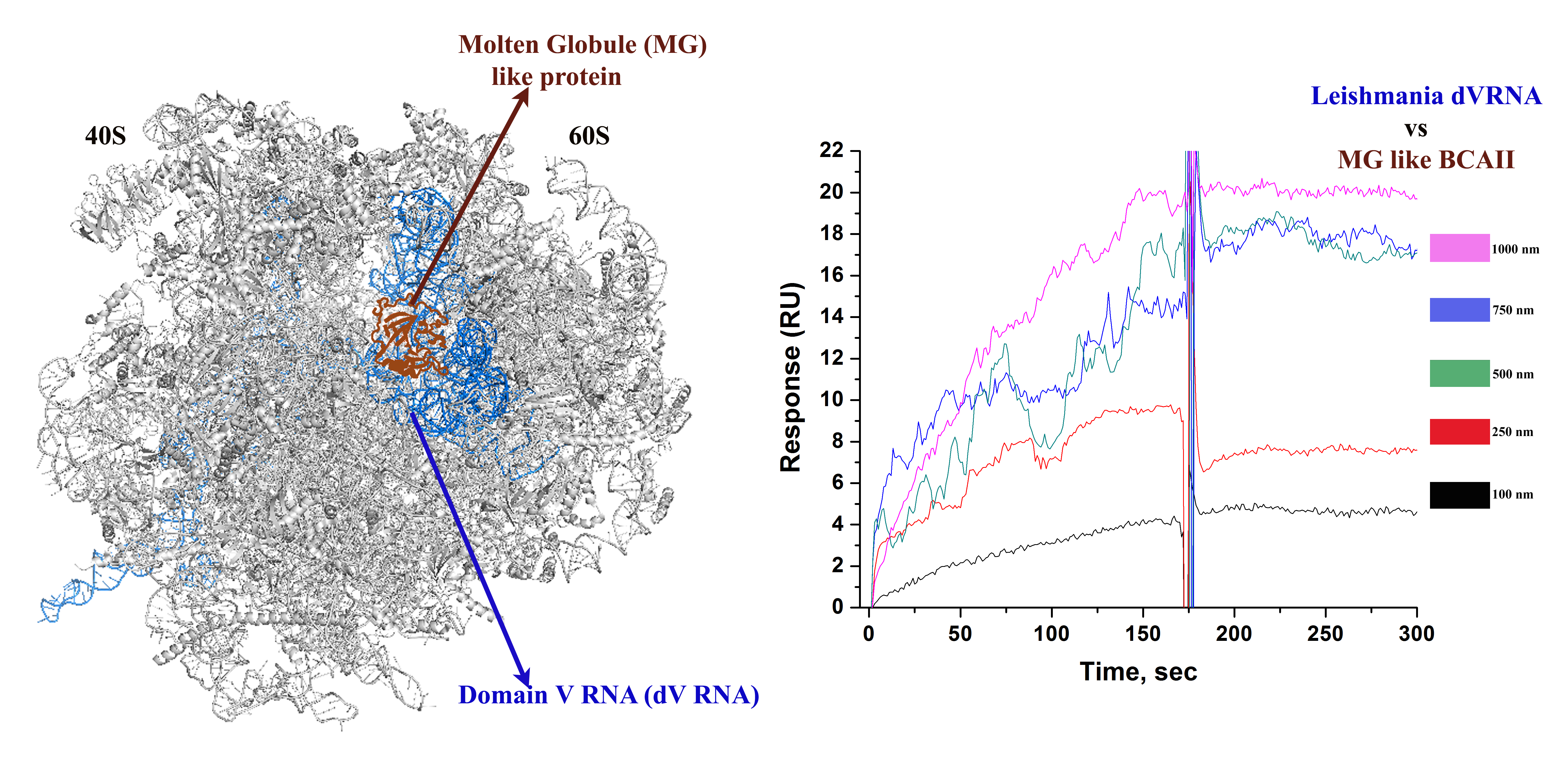

Supplement: S1 Graphical Abstract — (TIF) [file pone.0153928.s001.tif]
