# Supplementary material for: Mechanistic Insight into the Reactivation of BCAII Enzyme from Denatured and Molten Globule States by Eukaryotic Ribosomes and Domain V rRNAs
Source: PLoS One. 2016 Apr 21;11(4):e0153928. doi: 10.1371/journal.pone.0153928 (PMC4839638; doi:10.1371/journal.pone.0153928)
Supplement: S1 Table — (PDF) [file pone.0153928.s002.pdf]

S1 Table. **Previous mutational studies done on *E. coli* domain V** to identify the bases responsible for the RNA-mediated folding activity of ribosome [32, 17; Das D *et al. J Biol Chem* 287: 37508-37521 (2012), Pang Y *et al. J Biol Chem* 288: 19081-19089 (2013)] and corresponding bases in the PTC regions of *L. donovani* and S. *cerevisiae* (the nucleotides different in other species compared to *E.coli* are marked in bold).

E.coli	*L.donovani*	*S.cerevisiae*
A2059	A	A
A2062	A	A
G2472	G	G
U2473	U	U
U2474	U	U
C2475	C	C
U2491	U	U
U2492	U	U
U2493	U	U
G2494	G	G
U2500	U	U
C2501	C	C
**C2551**	**U**	**U**
U2552	U	U
U2554	U	U
U2555	U	U
**A2560**	**C**	**C**
**U2561**	**A**	**A**
**U2562**	**C**	**C**
U2563	U	U
**A2564**	**G**	A
U2586	U	U
A2587	A	A
A2588	A	A
