# Supplementary material for: Mechanistic Insight into the Reactivation of BCAII Enzyme from Denatured and Molten Globule States by Eukaryotic Ribosomes and Domain V rRNAs
Source: PLoS One. 2016 Apr 21;11(4):e0153928. doi: 10.1371/journal.pone.0153928 (PMC4839638; doi:10.1371/journal.pone.0153928)
Supplement: S2 Table — (PDF) [file pone.0153928.s003.pdf]

S2 Table. Previous additional mutational studies done on yeast domain V [17; Pang Y *et al. J Biol Chem* 288: 19081-19089 (2013)] which showed involvement in the protein folding activity (the nucleotide different in *E.coli* compared to other species is marked in bold).

S.cerevisiae	*L.donovani*	*E.coli*
U2873	U	**$^{\Psi}$U2504**
G2874	G	G2505
U2875	U	U2506
C2876	C	C2507
U2954	U	U2585
