# Supplementary material for: Mechanistic Insight into the Reactivation of BCAII Enzyme from Denatured and Molten Globule States by Eukaryotic Ribosomes and Domain V rRNAs
Source: PLoS One. 2016 Apr 21;11(4):e0153928. doi: 10.1371/journal.pone.0153928 (PMC4839638; doi:10.1371/journal.pone.0153928)
Supplement: S3 Table — (PDF) [file pone.0153928.s004.pdf]

S3 Table. Percent Identity Matrix (Created by Clustal 2.1) showing *L. donovani* domain V rRNA similar sequence identity with *S. cerevisiae and H. Sapiens domain V,* where as less identity with *E. coli* domain V rRNA.

**Species vs. %sequence identity**	***E. coli***	***L. donovani***	***S. cerevisiae***	***H. sapiens***
E. coli	100	60.42	60.55	61.26
L. donovani	60.42	100	77.9	76.7
S. cerevisiae	60.55	77.9	100	81.6
H. sapiens	61.26	76.7	81.6	100
