# Supplementary material for: Mechanistic Insight into the Reactivation of BCAII Enzyme from Denatured and Molten Globule States by Eukaryotic Ribosomes and Domain V rRNAs
Source: PLoS One. 2016 Apr 21;11(4):e0153928. doi: 10.1371/journal.pone.0153928 (PMC4839638; doi:10.1371/journal.pone.0153928)
Supplement: S1 Fig — (A) E. coli 70S crystal structures (PDB codes: 2I2U, 2I2V) showing domain V of 23S rRNA in brown colour, and nucleotides responsible for denatured protein binding as identified in [Das A et al. J Biol Chem 286: 43771-43781(2011)] are coloured in navy blue. Entrance of the polypeptide tunnel is marked. (B) Available Domain V structures from different organisms (PDB codes: E. coli, 2I2V; S. cerevisiae, 4V88; T. brucei, 4V8M) superimposed showing very less deviation (RMSD < 5Å). Land marks: CP, central protuberance; SB, P-proteins stalk base. (PDF) [file pone.0153928.s005.pdf]

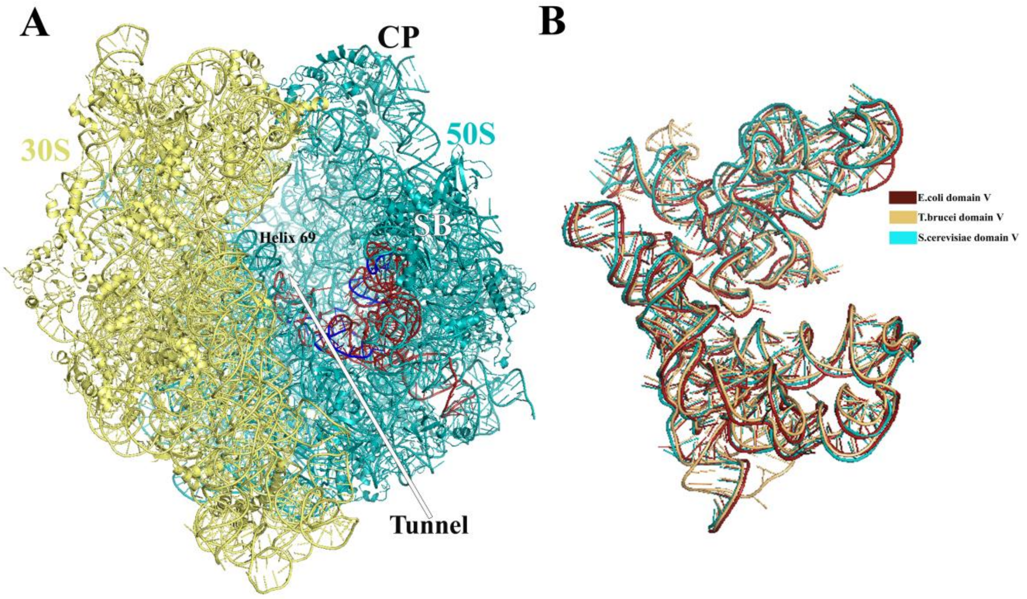

S1 Fig. Structural information on Domain V rRNA. (A) *E. coli* 70S crystal structures (PDB codes: 2I2U, 2I2V) showing domain V of 23S rRNA in brown colour, and nucleotides responsible for denatured protein binding as identified in [Das A *et al*. *J Biol Chem* 286: 43771-43781(2011)] are coloured in navy blue. Entrance of the polypeptide tunnel is marked. **(B)** Available Domain V structures from different organisms (PDB codes: *E. coli*, 2I2V; *S. cerevisiae*, 4V88; *T. brucei*, 4V8M) superimposed showing very less deviation (RMSD < 5Å).
Land marks: CP, central protuberance; SB, P-proteins stalk base.
